# Incidence and etiology of hemolytic-uremic syndrome in children in Norway, 1999–2008 – a retrospective study of hospital records to assess the sensitivity of surveillance

**DOI:** 10.1186/1471-2334-14-265

**Published:** 2014-05-16

**Authors:** Gaute Reier Jenssen, Eirik Hovland, Anna Bjerre, Hans-Jacob Bangstad, Karin Nygard, Line Vold

**Affiliations:** 1 Department of Infectious Disease Epidemiology, Norwegian Institute of Public Health (Nasjonalt Folkehelseinstitutt), Postboks 4404 Nydalen, Oslo NO 0403, Norway; 2 Faculty of Medicine, University of Oslo, Oslo, Norway; 3 Oslo University Hospital, Oslo, Norway

**Keywords:** Enterohaemorrhagic *E. coli* - EHEC, Epidemiology, Haemolytic uraemic syndrome, Surveillance, Shiga toxin producing *E. coli* - STEC

## Abstract

**Background:**

Public awareness of hemolytic-uremic syndrome (HUS), especially related to Shiga toxin-producing *Escherichia coli* (STEC), has increased in Europe in recent years; accentuated in Norway by a national outbreak in 2006 and in a European context especially by the 2011 outbreak originating in Germany. As STEC surveillance is difficult due to diagnostic challenges in detecting non-O157 infections, surveillance of HUS can be used to indicate the burden of STEC infection. Until 2006, notification of HUS to the Norwegian Communicable Disease Surveillance System (MSIS) was based on microbiologically confirmed infection with enterohemorrhagic *Escherichia coli* (EHEC), humanpathogenic STEC. In 2006, diarrhea-associated HUS (D^+^HUS) was made notifiable based on clinical criteria alone. The incidence and etiology of HUS in children in Norway has not previously been described.

**Methods:**

In order to assess the sensitivity of STEC and D^+^HUS surveillance and describe the incidence and etiology of HUS in children in Norway, we conducted a nationwide retrospective study collecting data from medical records from pediatric departments for the period 1999–2008 and compared them with data from MSIS. Descriptive statistics are presented as proportions, median, average and mean values with ranges and as incidence rates, calculated using population numbers provided by official registries.

**Results:**

Forty-seven HUS cases were identified, corresponding to an average annual incidence rate of 0.5 cases per 100,000 children. Diarrhea-associated HUS was identified in 38 (81%) cases, of which the median age was 29 months, 79% were <5 years of age and 68% were girls. From 1999 to 2006, thirteen more diarrhea-associated HUS cases were identified than had been notified to MSIS. From the change in notification criteria to 2008, those identified corresponded to those notified. STEC infection was verified in 23 (49%) of the HUS cases, in which O157 was the most frequently isolated sporadic serogroup.

**Conclusions:**

Our results show that the incidence of HUS in children in Norway is low and suggest that D^+^HUS cases may be underreported when notification requires microbiological confirmation. This may also indicate underreporting of STEC infections.

## Background

Hemolytic-uremic syndrome (HUS) is a clinical syndrome characterized by microangiopathic hemolytic anemia, impaired renal function and excessive platelet consumption leading to thrombocytopenia [1]. HUS is considered to be the most common cause of acute kidney injury (AKI) in European children, mainly affecting pre-school-aged children [2-4]. In the long term, HUS is associated with complications such as hypertension and end-stage renal disease with a death rate of approximately 3–5% [3]. Based on clinical presentation and probable etiology, HUS is commonly divided into diarrhea-associated HUS (D^+^HUS) and non-diarrhea-associated HUS (D^−^HUS), also called atypical HUS. Recent classifications have suggested a more specific approach based on causality and clinical associations [2]. Usually, around 90% of cases are D^+^HUS, while 10% are atypical HUS [5-9].

The most common cause of D^+^HUS in children is infection with Shiga toxin-producing *Escherichia coli* (STEC) [1], although not all STEC-associated HUS (STEC-HUS) cases present with diarrhea [7,10-12]. Shiga toxins are produced and released by STEC bacteria and are the main cause of STEC-HUS [1]. There are two main types of Shiga toxins, Shiga toxin 1 and Shiga toxin 2 (Stx 1 and 2). The latter is more frequently found in bacteria causing HUS than Shiga toxin 1. Ruminants are the main reservoir for STEC and infections are mainly food or waterborne. In D^−^HUS, the most common causes are infection caused by *Streptococcus pneumoniae* (SP-HUS) and genetic forms of HUS [9].

HUS and STEC infections are under epidemiological surveillance in the EU. In 2012, the European Commission updated the European case definition for STEC, requiring laboratory confirmation of Stx or *stx* gene(s), except when STEC O157 is directly isolated [13]. Surveillance of HUS and STEC in many countries is based on this case definition, requiring laboratory confirmation prior to notification. There are certain challenges in the surveillance of STEC. Far from all STEC infections are treated by a physician, identification of the infectious agent in mild infections might be judged unnecessary by clinicians and until more recent years, verification of non-O157 serogroups was difficult. Because of this, some countries, including France, have previously used surveillance of HUS to follow trends and identify outbreaks of STEC infection [14]. This is based on STEC being the causal factor in most HUS cases. In 2009, 3573 cases of STEC were reported in the EU, of which half were caused by serogroup O157 [4]. In Europe and America, this serogroup is most frequently isolated from HUS patients [2,4,6,12,15], while O111 is dominant in Australia [16]. Serotype O157:H7 is the most easily diagnosed serotype, based on its failure to ferment sorbitol within 24 hours of incubation [17,18]. However, in recent years, changes in diagnostic procedures have led to the isolation of an increasing variety of non-O157 STEC serogroups from HUS patients in Europe, including O26, O91, O103, O111, O113, O121, O128, O145 and sorbitol-fermenting O157 (SF O157) [4,7,8,14,19]. Recent outbreaks of STEC infection in Europe, with varying degrees of HUS development, have resulted in increased awareness. In 2011, *E. coli* O104 caused a large outbreak with many HUS cases in Germany. While the majority of cases occurred among adults, it also affected more children than previously seen in any other European outbreak to date [20]. In Norway, a national outbreak of STEC O103:H25 occurred in 2006, in which nine children developed HUS, including one with fatal outcome [21,22], raising awareness among physicians as well as microbiological laboratories.

Notification of EHEC infections to the Norwegian Surveillance System for Communicable Diseases (MSIS) has been mandatory since 1989 [18]. In Norway, all clinicians and microbiological laboratories analyzing human specimens are required by law to notify cases of group A infectious diseases, such as STEC infection, to MSIS at the Norwegian Institute of Public Health. Prior to the Norwegian outbreak in 2006, notification was only required for HUS cases with laboratory-confirmed STEC infection. In December 2006, after the outbreak, notification criteria were changed to include all D^+^HUS cases, based solely on clinical presentation. Since microbiological confirmation of STEC-HUS takes on average 14 days (measured from the day the stool sample is taken to the day the case is reported), the change in notification criteria aimed to improve the timeliness of reporting. In addition, clinically-based notification criteria were expected to increase the sensitivity of the surveillance system, as detection of STEC in a stool sample can be difficult and the bacteria may not be present at the time a patient develops HUS. Measures to improve diagnostic procedures were also implemented after the 2006 outbreak, and the laboratories methodology was gradually expanded to include PCR screening for presence of *stx* at all the microbiological laboratories. After implementation, ring tests sent out to all laboratories in the following years showed improved diagnostic capabilities for non-O157 serogroups.

The main aim of this study was to determine the annual incidence of D^+^HUS among children <16 years of age diagnosed in Norway from 1999 up to and including 2008 through a review of medical records, compare these numbers with cases reported to MSIS, and consequently assess the sensitivity of the D^+^HUS surveillance. The secondary aim was to describe annual incidence and etiology of all types of HUS in the same age group and for the same time period.

## Methods

### Design and data collection

We performed a retrospective, descriptive study. Data were collected from medical records from 24 pediatric departments of Norwegian hospitals from patients <16 years of age admitted from the 1^st^ of January, 1999, to the 31^st^ of December, 2008. All hospitals with capacity and competence for supportive care of HUS and/or AKI patients were included.

Potential cases were identified by performing medical record searches for pediatric patients tagged with ICD-10 codes D59.3 (HUS), N17 (AKI) and/or N00/N01/N05 (acute nephritic syndrome/rapidly progressive nephritic syndrome/unspecified nephritic syndrome). Apart from D59.3, the diagnostic codes were investigated to identify potentially misdiagnosed cases of HUS. Only cases matching the case definitions were included, regardless of ICD-10 code. Medical records were assessed in both electronic and paper form. Data were registered in forms made in EpiData (http://www.epidata.dk), which were designed through a pilot project to determine the availability of desired variables in standard medical records. Data files were encrypted according to the information security standards of the Norwegian Institute of Public Health. Data on cases of HUS and STEC notified from the 1^st^ of January, 1999, to the 31^st^ of December, 2008, were exported from MSIS. Population figures for children <16 years were acquired from Statistics Norway (SSB).

### Case definitions

A hemolytic-uremic syndrome (HUS) case was defined as:

a case clinically compatible with all the following laboratory findings of

o thrombocytopenia (<150 × 10^9/L)

AND

o anemia (Hgb < 10.5 g/dL)

▪ of hemolytic origin, with elevated serum LD (>500 U/L)

AND

o acutely reduced renal function (serum creatinine >35 μmol/L for patients < 1 years of age, > 80 μmol/L for patients 1-15 years of age)

AND

o Either

▪ reported presence of fragmented red blood cells (schiztocytes) on peripheral blood smear; a sign of microangiopathic changes consistent with hemolysis, an important part of HUS pathophysiology [1]

OR

▪ if peripheral blood smear was missing in the journal; probable clinical HUS confirmed by consulting a clinician with expertise in pediatric nephrology.

A diarrhea-associated HUS (D^+^HUS) case was defined as a HUS case with either:

– a clinical presentation of prodromal diarrhea, without verifiable causative etiology (probable STEC-HUS).

or

– STEC-HUS, defined as a HUS case with laboratory-verified STEC-infection.

A D^-^HUS case was defined as any non-diarrhea-associated HUS cases of non-STEC causality.

### Microbiology

Information on microbiological findings was gathered from medical records and from MSIS for the notified cases. MSIS receives data on microbiological characteristics from the regional laboratories as well as from the National Reference Laboratory for Enteropathogenic Bacteria in Norway.

### Statistical analysis

Calculations were performed using Microsoft Excel. Descriptive statistics are presented as proportions, median, average and mean values with ranges and as incidence rates, calculated using population numbers provided by official registries.

### Ethical considerations

The study was approved by the Regional Ethical Committee South East A. Dispensation was granted from patient confidentiality regulations as potential participants would only be identifiable following the review of medical records. It was therefore not necessary to contact all potential cases to gain their consent prior to collecting journal data. However, once cases were identified through the medical journal review process, the parents were notified and could elect to withdraw from the study. No patients chose this option.

## Results

### Sensitivity of the D^+^HUS and STEC surveillance

**Figure 1:**
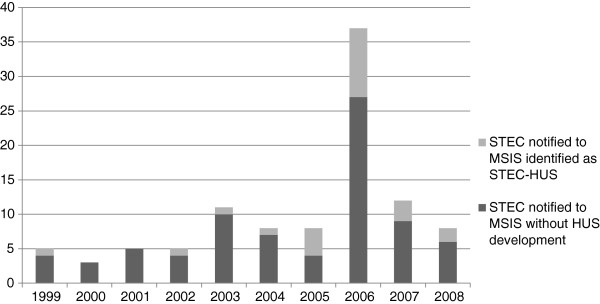
**HUS associated with STEC cases in children in Norway notified to MSIS between 1999 and 2008.** Cases of shiga toxin producing *E. coli* (STEC) infection reported to the Norwegian Communicable Disease Surveillance System, with share of cases identified in medical records as associated to hemolytic-uremic syndrome (HUS) in children <16 years of age, Norway, 1999–2008 (n = 102).

In the period 1999 up to and including 2008 28 HUS cases among children <16 years of age were notified to MSIS. Three cases, that is one case registered twice (two different hospitals) in 2003, and one in 2007 were identified and excluded from this study as they were initially admitted to a hospital abroad. In the same period, 102 cases of STEC infection were notified in the same age group. We identified 23 cases of STEC-HUS in medical records in the study period (Figure 1). Accordingly, 23% of the STEC-cases notified to MSIS in the period were cases with HUS.

Twenty of the HUS cases in MSIS were notified from the start of the study period up to and including 2006, and five after 2006. 17 of the cases notified before 2007 were identified as STEC-HUS cases; the remaining three were identified as probable STEC cases. These three were admitted to hospital just before and after the outbreak in 2006, thus probably notified as potential outbreak cases. The five cases notified after 2006 were identified STEC-HUS cases. The remaining STEC-HUS case, from 2005, was not notified to MSIS. The corresponding numbers identified in the medical records search were 33 and five for the period 1999–2006 and 2007–2008, respectively (Table 1).

**Table 1:** Diarrhea-associated HUS cases in children in Norway notified and identified in children in Norway

**Identified by/Year**	**1999-2006**	**2007-2008**	**Total**
MSIS	20	5	25
Medical records	33	5	38
Proportion reported to surveillance	61%	100%	66%

Cases of diarrhea-associated hemolytic-uremic syndrome in children <16 years of age reported to the Norwegian Communicable Disease Surveillance System (MSIS) and identified through medical record search, Norway 1999–2008.

### Incidence and etiology of all types of HUS in children 1999–2008

**Figure 2:**
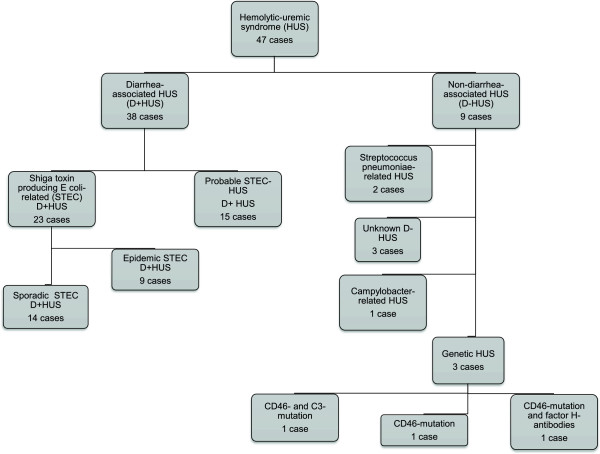
**Etiology of HUS in children in Norway.** Etiological distribution of cases of hemolytic-uremic syndrome (HUS) in children <16 years of age, Norway, 1999–2008 (n = 47).

**Figure 3:**
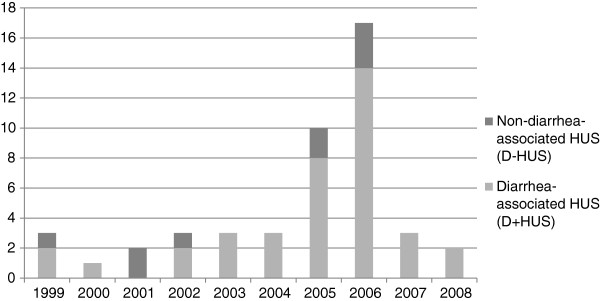
**Yearly occurrence of HUS in children in Norway between 1999 and 2008.** Yearly occurrence of cases of hemolytic-uremic syndrome (HUS) in children <16 years of age, Norway, 1999–2008 (n = 47).

Based on information from the medical records, a total of 47 cases of HUS in children were identified from 24 different Norwegian hospitals in the period 1999 to 2008 (Figures 2 and 3), varying from one case (in 2000) to 17 cases (in 2006) per year (Figure 3). Of the 47 HUS cases, 44 had the diagnostic code D59.3 (HUS). Three cases, two probable STEC-HUS and one SP-HUS, were identified as HUS through the diagnostic code for acute kidney injury (AKI); N17. These were all recognized as HUS in the journal, but had been given the wrong diagnose code. All three fit the inclusion criteria.

The average annual incidence rate of HUS of any etiology was estimated to be 0.5 cases per 100,000 children (range; lowest and highest year, respectively; 0.1-1.8). Thirty-one (66%) were female. The incidence rate was highest in children <5 years of age, with an estimated average annual incidence rate of 1.3 cases per 100,000 children (range; lowest and highest year, respectively; 0.0-3.8) (Table 2). The highest proportion of cases was in children aged one year, accounting for 34% of the cases (Table 2). The median age at initial admission was 29 months (range, 5 months-15 years).

**Table 2:** Epidemiology of HUS in children in Norway between 1999 and 2008

**Type of HUS**	**STEC-HUS**	**Probable STEC-HUS**	**Total D+HUS**			**D-HUS**		**All HUS**		
**Measure**	**Cases (N)**	**Cases (N)**	**N**	**%**	**IR**	**N**	**%**	**N**	**%**	**IR**
**Age**										
0-4 y	19	11	30	79	1.0	8	89	38	81	1.3
0 y	2	1	3	8	0.5	2	22	5	11	0.9
1 y	7	5	12	32	2.1	4	44	16	34	2.7
2 y	5	3	8	21	1.4	0	0	8	17	1.4
3 y	0	2	2	5	0.3	0	0	2	4	0.3
4 y	5	0	5	13	0.8	2	22	7	15	1.2
5-9 y	3	3	6	16	0.2	1	11	7	15	0.2
10-15 y	1	1	2	5	<0.1	0	0	2	4	<0.1
Total	23	15	38	100	0.4	9	100	47	100	0.5

Age-specific distribution (in number), proportion (in percentage) and incidence rate (IR; in average annual incidence rate in cases per 100,000 children) for diarrhea-associated (D^+^HUS), with and without laboratory identified STEC infection (probable STEC-HUS) and the two combined, non-diarrhea-associated (D^−^HUS) and all of the cases of hemolytic-uremic syndrome (all HUS) in children in Norway, 1999–2008 (N = 47).

Based on clinical presentation, cases were categorized into 38 (81%) D^+^HUS cases and 9 (19%) D^−^HUS cases (Figure 2). In the medical records, results from stool examination were available for 43 (91%) patients and for serological testing for 28 (60%) patients. STEC infection was detected in 22 (51%) of the stool samples and seven (25%) of the serological samples. One or both of these tests were performed in 44 of the cases, and STEC infection was confirmed in 23 (52%) of them, thus in one case STEC was only detected by serological testing.

Of the 38 D^+^HUS cases, 29 (76%) were sporadic and nine (24%) were outbreak cases; all nine cases were from the 2006 outbreak. The estimated average annual incidence rate for D^+^HUS was 0.4 per 100,000 children (range; lowest and highest year, respectively; 0.0-1.4) (Table 2). Twenty-six (68%) were female. The estimated average annual incidence rate for D^+^HUS was highest among children <5 years of age with 1.0 per 100,000 children (range; lowest and highest year, respectively; 0.0-3.5). This group constituted 30 (79%) of the D^+^HUS cases. STEC was confirmed in 23 (61%) of the 38 D^+^HUS cases. The remaining 15 cases presented with diarrhea, but without verified STEC infection or etiology and thus classified as probable STEC-HUS. In these cases, follow-up was evaluated in available medical records for a minimum of 1.5 years from first hospital admittance; none experienced recurrence of their HUS during this time.

The distribution of serotypes of STEC isolated from the 23 laboratory confirmed cases, is shown in Table 3. Excluding nine O103 cases from the 2006 outbreak [21,22], O157 was the most common serogroup involved in sporadic D^+^HUS, found in five cases (36%). The remaining nine (64%) sporadic cases were non-O157. *Stx* presence was reported in 12 (52%) of the 23 STEC-HUS cases, with *stx2* present in 10 cases and both *stx1* and *stx2* in two cases (Table 3). In the 11 cases where no *stx* was found, the strains isolated from the patients were considered STEC that had lost their toxin coding genes. Four of these strains were isolated from outbreak cases, and Multiple-locus variable-number tandem-repeats analysis (MLVA) genotyping of the strains was used to categorize these as the causative agent, even if they were *stx* negative [23].

**Table 3:** Serology of STEC-related HUS in children in Norway between 1999 and 2008

**Case type – toxin type**	**Sporadic**	**Epidemic**	**Shiga-like toxin 1**	**Shiga-like toxin 2**	**Both shiga like-toxin 1 and 2**
**Serotype**					
O26: H11	1				1
O26: H?	1			1	
O87: H?	1				
O103: H25	0	9		5	
O103: H?	2				
O145: H25	1				
O145: H?	1				
O157: H7	2			2	
O157: H?	3			2	
Non-O103/O157	2				1
Total	14	9	0	10	2

Distribution of serotype (O: cell wall antigen number, H: flagella antigen) and shiga-like toxin profile in sporadic and epidemic cases of shiga toxin producing *E. coli*-related hemolytic-uremic syndrome in children in Norway, 1999–2008 (N = 23).

### D^−^HUS/atypical HUS

Of the 47 identified HUS cases, 9 (19%) were considered D^−^HUS. Five were male and four were female. Average annual incidence rate was <0.1 per 100,000 children (range; lowest and highest year, respectively; 0.0-0.3) (Table 2). Eight (89%) of the nine children were <5 years of age, and the last case was nine years. Two cases were related to pneumococcal infection (SP-HUS) and three were of genetic origin. All three patients had CD46-mutations. One had an additional C3-mutation and another had antibodies to factor H. In one case, *Campylobacter* was isolated and specified as causative in the medical record, without prodromal diarrhea*.* The remaining three were non-diarrhea-associated cases with unknown etiology (Figure 2).

## Discussion

In the period 1^st^ of January, 1999, to the 31^st^ of December 2008, we identified a total of 47 cases of HUS in children <16 years of age, with an estimated average annual incidence rate of 0.5 cases per 100,000 children. Of these cases, 81% were diarrhea-associated HUS (D^+^HUS), though only 61% of these were laboratory verified with a Shiga toxin-producing *Escherichia coli* (STEC) infection. We also found that before mandatory notification criteria were changed from D^+^HUS with laboratory verified STEC infection to clinical D^+^ HUS in December 2006 [18], only 61% of D^+^HUS cases were notified. After the case definition was amended, the number of cases notified to MSIS corresponds with the number of cases we found when systematically reviewing all patient medical records with relevant ICD-10 codes.

We assume that all D^+^HUS cases reported via MSIS are caused by STEC, since this is internationally recognized to be the most common etiological agent in HUS cases [12,18]. All D^+^HUS cases in our study are therefore coded as STEC cases. However, only 61% of the D^+^HUS cases found through our review of medical records had laboratory-verified STEC. This may reflect that the HUS cases were caused by other etiological agents causing D^+^HUS that we were not able to recognize, but it is more likely due to problems with diagnosing STEC in stool samples from HUS patients. HUS typically develops as a complication of STEC-related diarrhea, but patients often no longer have diarrhea when they develop HUS and may have stopped shedding the bacteria [1]. In addition, if the patient has non-O157:H7 STEC, the diagnostic methods are more complicated than if STEC is caused by O157:H7, allowing the etiological agent to be overlooked. This is especially true for cases occurring before the outbreak in 2006, as during that period many laboratories still based their diagnostics on cultivation and could easily miss the diagnosis [18]. In our study, we found that 64% of verified STEC cases were non-O157 when the outbreak-related STEC-HUS cases are excluded. However, O157 was the most frequently isolated serogroup causing sporadic HUS in Norway, as has been found in other European countries [6,7,10,11,14], South America [24,25] and North America [15,26].

Based on surveillance data from MSIS, an estimated 23% of children with STEC infections developed HUS, which is high in comparison to other countries; certain studies have shown that 10%-15% of children infected with STEC O157 develop HUS [6,12,26,27]. According to the European Center for Disease Prevention and Control, this proportion is about 8% [3]. However, these studies are based predominantly on O157 STEC cases. In the 2012 Germany outbreak, where a particularly aggressive strain of *E. coli* O104 was the cause, 22% of adults and a slightly higher proportion of children (approximately 24%) developed HUS [20]. The high proportion of HUS cases reported via MSIS may be explained by either an overestimation of HUS cases, an underreporting of STEC cases, or that STEC in Norway may be more likely to cause HUS than what is described for O157 in the literature. The first explanation is unlikely; in addition to the 23 confirmed STEC-HUS cases, we also found 15 probable STEC-HUS, all with classical clinical presentation of STEC-HUS. When considering the difficulties described for laboratory identification of non-O157:H7 STEC strains especially, it is probable that several of these are actually STEC-HUS cases. It is more likely that this high STEC-HUS/STEC-ratio is due to underreporting of STEC cases. Mild cases of STEC infection may only present with diarrhea, not requiring medical attention or submission of stool samples. Only severe cases, which are more likely to be complicated by HUS, are investigated thoroughly for a source. Isolation of STEC in D^+^HUS cases is also often dependent on stool samples being examined early in the disease progression [18]. Additionally, the difficulties in identifying non-O157:H7 STEC strains may result in several cases being missed, despite samples being taken and analyzed. In Norway, most of the cases are caused by non-O157:H7 STEC, and the virulence of non-O157:H7 strains is probably variable. Some strains, like sorbitol-fermenting O157, are now considered more likely to cause HUS than O157:H7 [28], whereas others might be less likely to cause HUS.

Although the proportion of STEC cases developing HUS was high, the overall incidence rate of HUS reported in our study is low compared to similar studies from other European countries, [6-8,11,14], likely due to a low incidence of diarrhea-associated HUS, since this accounts for the majority of HUS in children (81%). This again points to the low amount of STEC cases identified in this group and the study as a whole. A possible explanation for this may be the low prevalence of STEC among ruminants in Norway. In particular, surveys in sheep and cattle have found a low prevalence of O157 [18,29-31].

Our study also describes the burden and etiology of atypical HUS in children. As there is no mandatory notification of D^−^HUS cases in Norway, these cases were only identified and described after our search through medical records. Only nine D^−^HUS cases were identified in the ten year period, accounting for less than 20% of the total HUS burden in children. Of these, two were related to pneumococcal infection (SP-HUS), three were of genetic origin, one had a suspected associated with campylobacter infection and three were of unknown etiology. It is noteworthy that there were only two SP-HUS cases during the ten year period, as certain studies have indicated that this is an increasing problem globally [32,33].

There are some limitations to our study. As it is retrospective, it reflects the judgments made by clinicians several years ago, when HUS was relatively uncommon. A lack of awareness could have led to misdiagnosed cases. However, to minimize this we also searched for HUS cases in medical records where patients were diagnosed as acute kidney injury. We thereby identified three HUS cases without a HUS ICD-10 code added by the clinicians, instead coded only as acute kidney injury. Failure to detect STEC in HUS patients due to late sampling and diagnostic problems in the laboratory are also limiting factors in the study, as the etiology was not found in a notable proportion of cases.

## Conclusions

Our findings indicate that the occurrence of HUS, although low compared to other European countries, and STEC in Norway is higher than previously assumed. While we have no apparent explanation as to why the incidence of HUS is low, a possible contributing factor might be that the prevalence of STEC is low among ruminants, a known source of infection. The diagnostics have improved after the outbreak in 2006. Despite this, the results reinforce that clinicians should perform early stool sampling in HUS cases where STEC infection is suspected. Therefore, our recommendation is to reinforce the mandatory notification and surveillance of both D^+^HUS and laboratory verified STEC-infections and to further develop laboratory verification techniques of emerging non-O157 STEC serotypes.

Our study also illustrates that the proportion of laboratory verified HUS cases might be low. This highlights the need for surveillance based on clinical HUS without the need for laboratory confirmation.

## Competing interests

The authors declare that they have no competing interests.

## Authors’ contributions

GRJ led the writing of the manuscript and performed the statistical work. All authors contributed to the writing and reviewing of the article. LV, KN, EH and GRJ participated in the methodological and structural design of the study. AB, HJB, EH and GRJ participated in the design of the clinical aspects of the study. All authors have contributed in modifying and improving the study design throughout the process. GRJ and EH performed the nationwide collecting of study data, in which AB and HJB functioned as clinical consultants. All authors read and approved of the final manuscript.

## Pre-publication history

The pre-publication history for this paper can be accessed here:

http://www.biomedcentral.com/1471-2334/14/265/prepub
